# Antimicrobial efficacy of Kerr pulp canal sealer (EWT) in combination with 10% amoxicillin on *Enterococcus faecalis*: A confocal laser scanning microscopic study

**DOI:** 10.12688/f1000research.132047.1

**Published:** 2023-06-21

**Authors:** Madhureema De Sarkar, Kundabala Mala, Suchitra Shenoy Mala, Shama Prasada Kabekkodu, Srikant Natarajan, Neeta Shetty, Priyanka Madhav Kamath, Manuel Thomas

**Affiliations:** 1 Department of Conservative Dentistry and Endodontics, Manipal College of Dental Sciences, Mangalore, Manipal Academy of Higher Education, Manipal, Karnataka, 575001, India; 2 Department of Microbiology, Kasturba Medical College, Mangalore, Manipal Academy of Higher Education, Manipal, Karnataka, 575001, India; 3 Department of Cell and Molecular Biology, School of Life Sciences, Manipal Academy of Higher Education, Manipal, Karnataka, 576104, India; 4 Department of Oral Pathology and Microbiology, Manipal College of Dental Sciences, Mangalore, Manipal Academy of Higher Education, Manipal, Karnataka, 575001, India

**Keywords:** Enterococcus faecalis, Pulp Canal Sealer (EWT), Amoxicillin, Confocal Laser Scanning Microscope, Biofilm, antimicrobial action, Obturaion, Eugenol

## Abstract

**Background: **Sealers with antimicrobial properties play an important role in endodontic therapy success especially against *Enterococcus faecalis* infection found in failed root canal therapy. Addition of antibiotic agents to endodontic sealers may show significant increase in their antibacterial properties both against anaerobic and aerobic microbes. The purpose of the present study was to evaluate antimicrobial efficacy of Kerr pulp canal sealer (EWT) in combination with 10% amoxicillin against *E. faecalis* and post-root canal treatment viability of *Enterococcus faecalis *on the first and seventh day.

**Methods: **A total of 60 extracted human mandibular premolar teeth were decoronated after initial decontamination with 1% NaOCl. Root length standardized to 12 mm. Canal instrumentation was done using ProTaper Universal file system till size F2 using 5.25% NaOCl. It was then infected with a pure strain of *E. faecalis* for a period of four days. Obturation was done using plain sealer, (n=30) and sealer-antibiotic combination, (n=30). Half of the teeth were sectioned at 24 hours (S, SA) and other half were sectioned seven days after obturation (S7, SA7). All samples were stained with SYTO9 and propidium iodide for imaging under Confocal Laser Scanning microscope. Statistical analysis was performed with the statistical software SPSS v. 17.0 (SPSS for Windows; SPSS Inc, Chicago, IL). Data was analysed using One Way ANOVA and *post hoc* Tukey test to determine statistical significance with p value < 0.01 considered significant.

**Results: **Statistically significant differences were observed in green to red ratio between group S (9.561976) and S7 (0.435418) (p < 0.01). There was no difference found between SA (mean of green to red ratio, (0.70431) and SA7 (mean of green to red ratio, 0.85184).

**Conclusions: **Antibiotics added to the sealer effectively eradicated of *E. faecalis* 24 hours post-obturation. However, after seven days, plain sealer was as effective as sealer-antibiotic combination.

## Introduction

Microorganisms, biofilm and irritants have been the principal causative elements associated with the pathogenesis and progression of pulp and periapical diseases. Eradicating them is the ultimate goal of endodontic therapy. This can be achieved through the combination of asepsis, chemical preparation mechanical preparation, antimicrobial irrigating solutions, intracanal medicaments and fluid tight seal of canal system by obturation. ^1^ In many studies, *Enterococcus faecalis* has been identified as the most common species associated with persistent or secondary intraradicular infections that do not respond to treatment. ^2^^–^^6^ It has been found that *E. faecalis* is isolated in 23-70% of positive cultures of obturated root canals that show signs of chronic apical periodontitis. ^2^^,^^7^^–^^12^ It is associated more with asymptomatic cases and exhibit widespread genetic polymorphisms and they often occur in monoculture. ^13^^,^^14^

Complete elimination of microbes from root canal system is prevented by difficulty in negotiating complex anatomy, role of dentinal fluid in reducing efficacy of irrigants and intracanal medicaments. Also, high microbial virulence, biofilm formation and relative antimicrobial resistance of infecting bacteria prevent canal disinfection. ^15^^–^^19^ In a biofilm, most antimicrobial agents and irrigants only act against microorganisms in its superficial layer, leaving those in the deeper layers unaffected. ^20^

Root canal treatment failures can be prevented or at least minimized by following proper irrigation and obturation protocols. The obturating materials and root canal sealers should exhibit anti-microbial properties, sustained over a period of time, to prevent bacterial growth. ^21^ These solid core obturating materials at times are unable to reach the irregularities of the root canal space such as accessory canals, apical ramifications, isthmuses, the fins, ramifications, and cul-de-sacs. Thus, root canal sealers are used in conjunction with these solid core obturating materials in order to fill these anatomical irregularities completely. The choice of a good sealer greatly affects the outcome of endodontic treatment. ^22^

Several anti-microbials are being added to improve antibacterial properties of sealers, including antibiotics. When conventional root canal treatment alone is insufficient, antibiotics such as penicillin and amoxicillin can be prescribed for treating endodontic infection. ^23^ Using five antibiotics, Hoelscher *et al.* found Kerr pulp canal sealer (PCS) EWT enhanced antibacterial activity against *Enterococcus faecalis.* ^24^ All of the sealer-amoxicillin combinations in the investigation displayed the highest zone of inhibition under both anaerobic and aerobic conditions. ^25^ Amoxycillin at 10% volume showed the best result as an additive to PCS (EWT) sealer with the least mean apical leakage which is clinically significant. ^26^

Antimicrobials within dentinal tubules have a greater antibacterial effect on the seventh day than it did at 24 hours according to study by Heling *et al.* ^27^Antimicrobial regimens can be evaluated during and post-treatment by culture from the site of infection, blood profile and powerful microscopic examination of the histopathological section. Recently developed Confocal Laser Scanning microscopy has gained popularity in the field of life sciences since it can be used to view and identify single cellular structures. It facilitates immediate fixation of live and dead bacteria, which is not possible in any culture-based method.

The present study was conducted to evaluate the antimicrobial efficacy of the addition of 10% amoxicillin to PCS (EWT) against *E. faecalis*, at 24 h and seven days following obturation, under Confocal Laser Scanning Microscope.

## Methods

### Preparation of dentin blocks

Prior to conducting the study, ethical clearance was obtained from Institutional Ethics Committee, Manipal College of Dental Sciences, Mangalore, Karnataka, India. Protocol approval with Ref. No. 15123. A total of 60 freshly extracted single rooted human premolars with Type I canal anatomy and mature root apex were selected and were stored in 1% sodium hypochlorite solution for 48 h for initial sterilization, and subsequently washed in sterile distilled water. Samples were decoronated to a length of 12 mm using a carborandum disc (Mani Inc. Japan) at a speed of 250 rpm attached to a slow speed handpiece (NSK Co., Japan) under water cooling. Canal orifices were enlarged with Gates Glidden drill size #5 (Mani Inc. Japan). Working length was determined by visual examination under 2.5X dental magnifying loupes (Pierson Surgical limited, Keeler, UK) using a 10 number K file (Dentsply-Maillefer, Ballaigues, Switzerland). All canals were then enlarged till size 25 hand K files using RC prep (Premier, India) for canal lubrication. Final preparation was done using rotary ProTaper Universal files (Dentsply, York, PA, USA, till File F2, using X-SMARTTM Electric Endo Motor. Throughout the instrumentation, 5.25% sodium hypochlorite was used to irrigate the canals, followed by irrigation with normal saline and 17% EDTA. Red nail varnish (Maybelline) was applied in double layers, to cover the outer surface of the samples except 3 mm from the apical end of roots. Samples were dried for 24 hours before being autoclaved (MELAG Euroklav 23 VS-S, Russia).

Infection of the dentin blocks: The *Enterococcus faecalis* strain ATCC 29212 (ATCC, USA) was cultured and reactivated in Brain Heart Infusion broth (BHI, Difco, Kansas City, MO, USA), which was maintained at 37°C for 24 hours. Broth was transferred to another BHI flask and incubated again for another 24 hours to achieve exponential growth and adjusted to McFarland standard No. 1 (3 × 108 CFU/mL).

On the first day, as described by Andrade FB, *et al.* ^28^ all dentinal blocks were infected over a period of 4 days. On the second day, following incubation, samples were agitated in a vortex for 10 s, and then inocula from the microtubes were discarded. One mL of sterilized BHI broth was inserted, following which a centrifuge cycle of 3,600 g for 5 min at 25°C was done. The microtubes were incubated again at 37°C for 24 hours. On the third day, a new inoculum of *E. faecalis* was inserted into the sample tubes, at exponential growth phase after seven hours of subculture in BHI broth. The centrifugation protocol was repeated twice at each speed, at 25°C. Procedures were repeated on the fourth day as described for the second day. On the fifth day, the samples were removed from the microtubes. Half of the samples were sectioned and then were stained with SYTO9 stain and the other half maintained in the incubator for another 7 days.

### Obturation

Sealer-antibiotic combination was prepared manually by mixing the antibiotic to the sealer 10% by weight using an electronic weighing scale (Sartorious Lab balance, Germany). ^29^ The sealer was mixed according to manufacturer’s specifications for obturation. Root canals were irrigated with 17% EDTA for one minute and dried with paper points (Diadent, S. Korea) Sealer-antibiotic paste was lightly coated on to the canal walls twice with gutta-percha and canals were obturated with gutta-percha points (Diadent, S. Korea) with lateral compaction technique. Access cavity was restored with composite resin (3M ESPE, USA). All specimens were incubated at 37°C in humid conditions.

Prior to obturation, the samples were randomly distributed into two groups of 30 specimens each. After the root filling, the samples were subdivided into two more groups of 15 teeth each, based on the time of assessment into the following:

Group 1: S - sealer group (24 h)

Group 2: SA - sealer antibiotic group (24 h)

Half if the samples sectioned on the seventh day were designated number ‘7’

Group 1 a: S7 - Seventh day sealer group

Group 2 a: SA7 - Seventh day sealer antibiotic group

Low-speed hand pieces with small round burs were used to fracture each cylindrical dentin block by making thin vertical grooves in the middle (Dentsply Maillefer, Ballaigues, Switzerland). Following this, specimens were fractured using a chisel and mallet into two semi cylindrical halves. The outer convex surface was ground using low speed handpiece (NSK Co., Japan) attached to a water cooler and a fine carbide bur (Dentsply-Maillefer, Ballaigues, Switzerland) at 300 rpm, to achieve a standard thickness of 1 mm. Thirty specimens from each experimental group were sectioned 24 hours post-obturation and the rest on the seventh day and stained for analysis under a confocal microscope.

### Sample preparation for confocal laser scanning microscopy

The sectioned dentin pieces were stained with SYTO 9 Green Fluorescent Nucleic Acid Stain (ThermoFisher Scientific, USA) and propidium iodide (Himedia, India), according to manufacturer’s instructions for 20 minutes in the dark at room temperature, and then rinsed with phosphate buffered saline for one minute. Each sample was then air-dried briefly and transferred into a micro centrifuge until used. Images were captured at magnification of 40×. In the case of SYTO9, the excitation/emission wavelengths were 480/500 nm, whereas in the case of propidium iodide, they were 490/635 nm. Image acquisition and analysis using CLSM at a resolution of 1024×1024 was carried out using Leica Application Suite (Leica Microsystems, Germany). Borders of the root canal were focused and two images per sample were obtained randomly. Background noise was then reduced in the images (Leica Application Suite software) and further quantitative analysis was carried out using ImageJ software (V ImageJ: Rasband, W.S., ImageJ, U. S. National Institutes of Health, Bethesda, Maryland, USA). ^30^^,^^31^

### Statistical analysis

One-way ANOVA and the Post hoc Tukey Test were used to analyse the data and establish its statistical significance. Significance was set at p < 0.05. The statistical programme SPSS v. 17.0 (SPSS for Windows; SPSS Inc, Chicago, IL) was used to conduct the analysis.

## Results

The results were interpreted as:

Green ratio was calculated by dividing the area of the living cells by the size of the entire magnified field of view at 40×.

Red ratio was calculated by dividing the area of the dead cells by the size of the entire magnified field of view at 40×.

Green to red ratio: the value obtained by dividing the green ratio with the red ratio.

**Figure 1.**
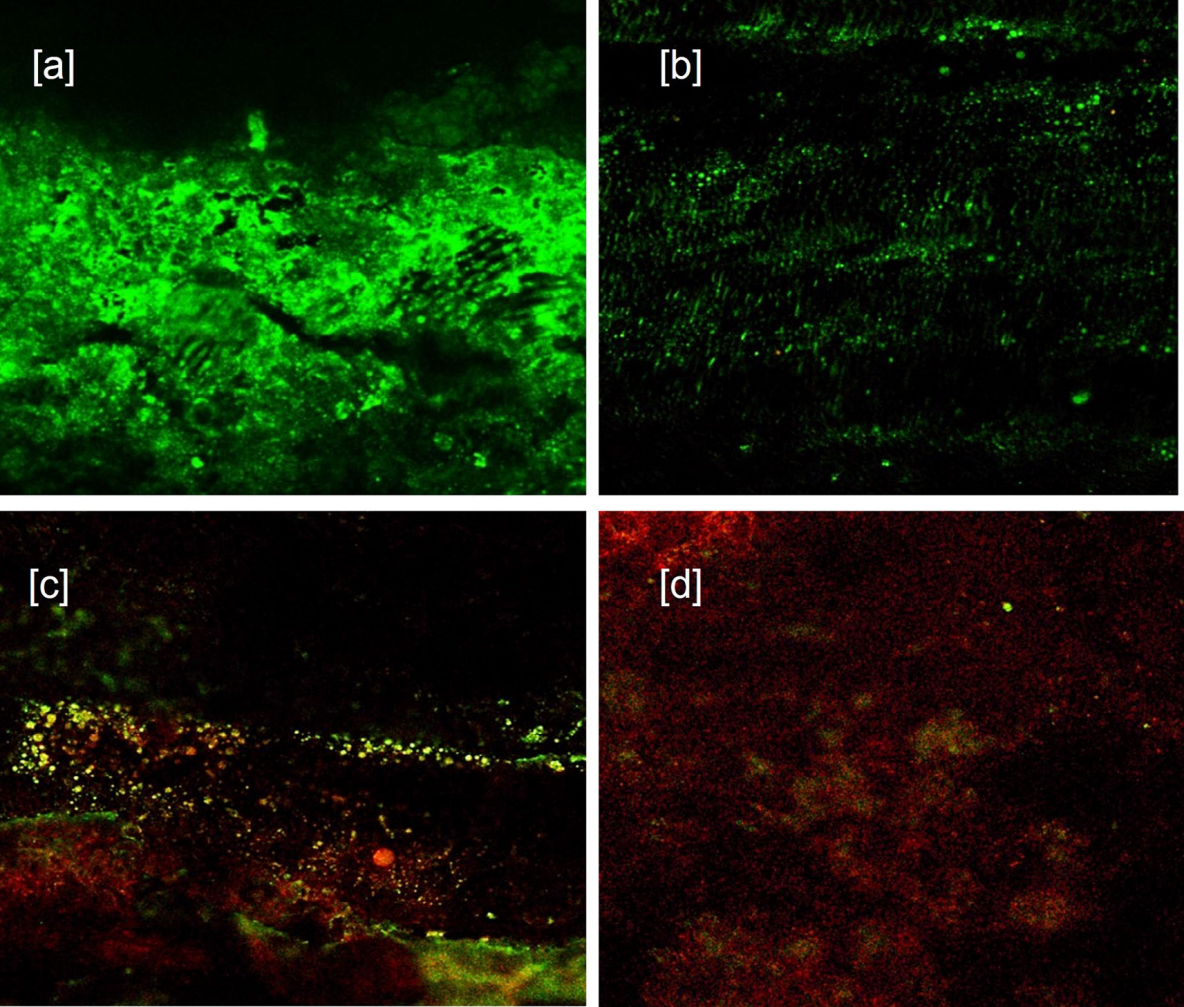
CLSM images (40×) for 24 hours groups showing. [a] Baseline for Sealer group. [b] Sealer group showing viable cells (in green color) at 24 hrs. [c] Baseline for Sealer-antibiotic group. [d] Sealer-antibiotic group showing viable cells at 24 hrs (in green color).

**Figure 2.**
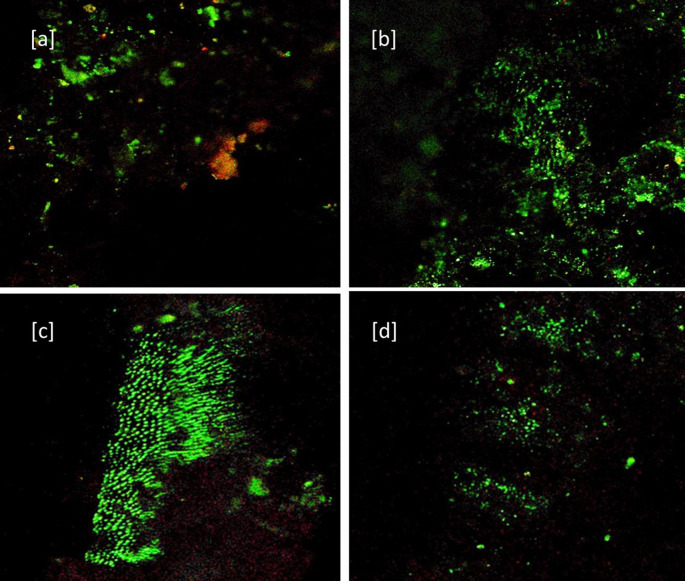
CLSM images (40×) after 7 days. [a] Baseline for Sealer group. [b] Sealer group showing dead cells (Red color) and viable cells (in green color). [c] Baseline for Sealer-antibiotic group. [d] Sealer-antibiotic group showing dead cells.

CLSM images (40×) for 24 hours groups showed: [a] Baseline for Sealer group [b] Sealer group showing viable cells (in green color) at 24 h. [c] Baseline for Sealer-antibiotic group [d] Sealer-antibiotic group showing viable cells at 24 h (in green color) ( Figure 1) After 7 days: [a] Baseline for Sealer group [b] Sealer group showing dead cells (Red color) and viable cells (in green color) [c] Baseline for Sealer-antibiotic group [d] sealer-antibiotic group showing dead cells ( Figure 2).

**Figure 3.**
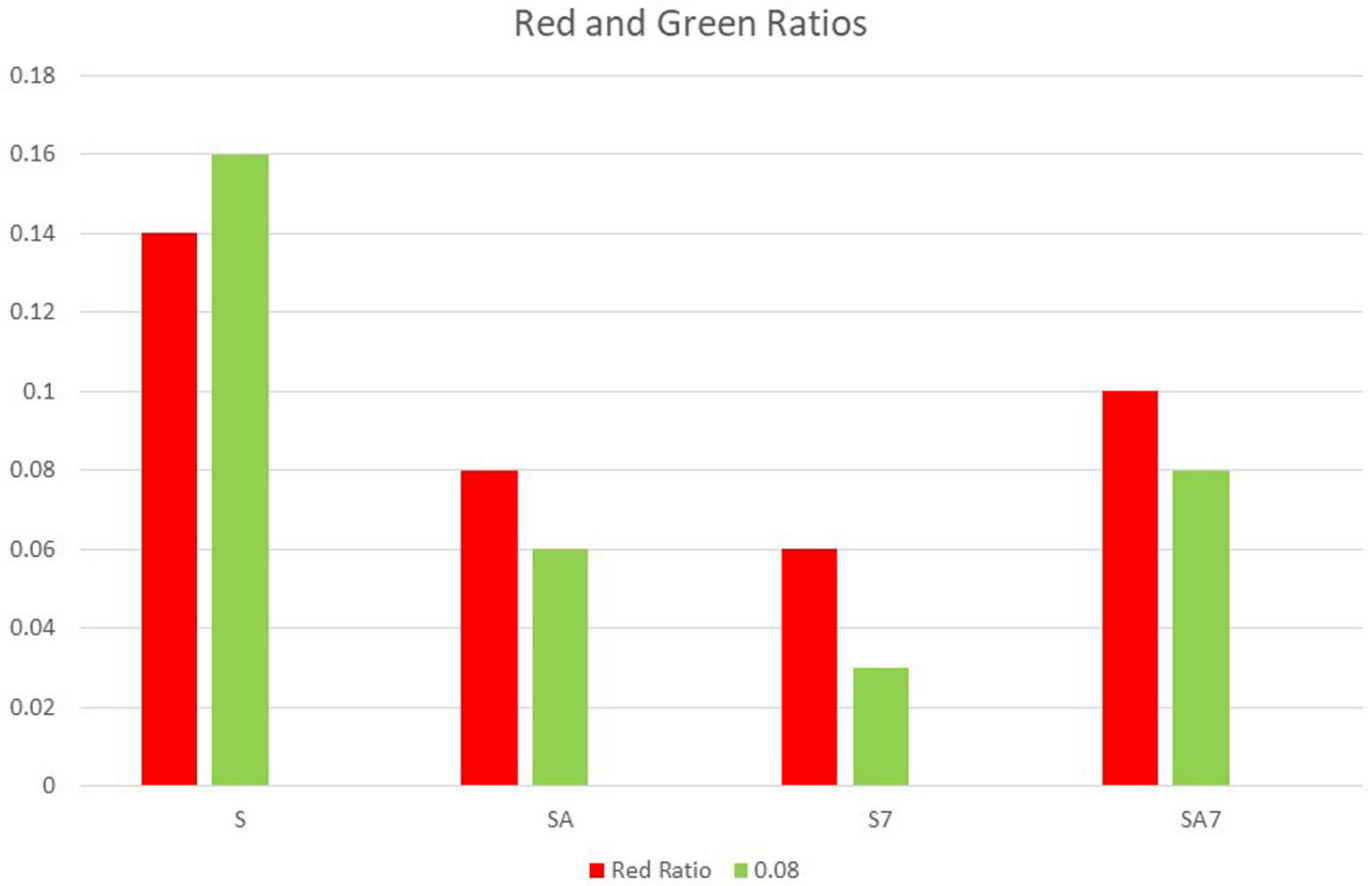
Bar graph shows individual Red and Green ratio of groups S7, SA7, SA, S.

**Figure 4.**
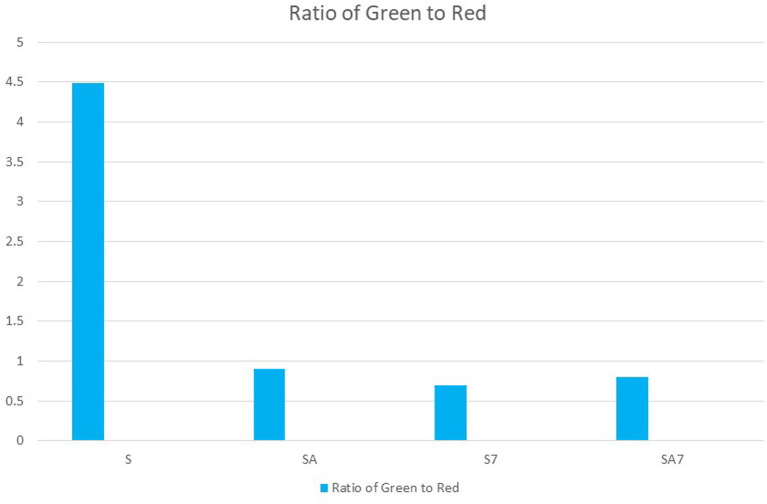
Bar graph shows Green to Red ratio of groups S7, SA7, SA, S. Bar graph showing in X axis green to red ratios of all groups. In Y axis showing different groups.

The first bar graph shows individual rd and green ratio of groups S7, SA7, SA, S ( Figure 3). The second bar graph showthe green to red ratios of all groups along the x axis, while they axis shows different groups ( Figure 4).

In the present study both, highest green and red ratio were seen in the S group followed by SA7, SA and least in S7 ( Figure 3). There was no statistical difference among the groups in red ratio, but green ratio of S group showed statistically significant difference with other groups ( Table 1). Ratio of green to red also shows that the mean value of S group was highest with lowest antibacterial effect on *E. Faecalis* followed by SA, SA7 and least in S7 with no statistically significant difference ( Figure 4). The ratios were comparable between SA and SA7 with a slightly better ratio in the SA group. The SA, SA7, S7 showed marked reduction in the green ratio and live bacteria when compared with the S group. Comparison of the red ratio using Post hoc Tukey test between different groups showed no statistically significant difference ( Table 2). Comparison of the green ratio using Post hoc Tukey test showed no statistically significant difference between different groups except between S7 and S showing significant reduction in live bacteria in S7 compared to S ( Table 2).

**Table 1.** One way ANOVA showing the difference in the mean between green ratio, red ratio, green to red ratio.

		N	Mean	Standard deviation	Statistics/mean squares	df2(welch) /F (ANOVA)	p value
**Red ratio**	S7	15	0.061117	0.073735	**0.018**	**1.006**	**0.398**
	SA7	15	0.108193	0.049143			
	SA	15	0.085111	0.107795			
	S	15	0.146622	0.235004			
	Total	60	0.101085	0.135374			
**Green ratio**	S7	15	0.032093	0.031729	4.874	26.179	**0.008**
	SA7	15	0.085349	0.056604			
	SA	15	0.061151	0.055771			
	S	15	0.167378	0.214539			
	Total	60	0.087357	0.123172			
**Ratio of Green to Red**	S7	15	0.70431	0.435418	0.99	26.103	0.413
	SA7	15	0.85184	0.520982			
	SA	15	0.905563	0.789528			
	S	15	4.492063	9.561976			
	Total	60	1.736525	4.943367			

**Table 2.** Post hoc Tukey test showing the mean difference between green ratio, red ratio, green to red ratio.

Dependent variable	Comparison group	Compared with	Mean difference	Standard error	p-value
**Red ratio**	S7	SA7	-0.0470768	0.049534	0.778
		SA	-0.0239944	0.053248	0.969
		S	-0.0855055	0.051159	0.349
	SA7	SA	0.0230823	0.051689	0.97
		S	-0.0384287	0.049534	0.865
	SA	S	-0.061511	0.053248	0.657
**Green ratio**	S7	SA7	-0.0532568	0.042269	0.592
		SA	-0.0290585	0.045438	0.919
		S	-.1352852*	0.043656	**0.016**
	SA7	SA	0.0241982	0.044108	0.947
		S	-0.0820284	0.042269	0.224
	SA	S	-0.1062266	0.045438	0.103
**Ratio of green to red**	S7	SA7	-0.1475299	1.759492	1
		SA	-0.2012524	1.891397	1
		S	-3.7877526	1.817196	0.172
	SA7	SA	-0.0537224	1.836027	1
		S	-3.6402227	1.759492	0.177
	SA	S	-3.5865002	1.891397	0.242

## Discussion

The S group (sealer without antibiotics at 24 hours) proved to have little antibacterial effect against *E. faecalis* at 24 hours compared to baseline. Even though highest red ratio is recorded for this group it could not outnumber the green ratio ( Figure 1 [a] Baseline, [b] after 24 h) The reason for the highest red ratio in S group could be due to increase in the free eugenol release from freshly mixed sealer or cytotoxicity expressed within the first few hours. This hypothesis is supported by *in vitro* study, which showed inhibition of *E. faecalis* only within the first five hours of incubation due to the release of free eugenol. ^32^ Eldeniz *et al*., also found that the antibacterial activity was higher at the 13th hour. Cytotoxicity of ZOE based sealers have been shown to sharply reduce after 24 hours of setting. ^33^ The green ratio was highest in this group when compared to other groups because of the decrease in the release of eugenol from set sealer at 24 hours, allowing *E. faecalis* to grow. The results are in agreement with Zhang *et al* who evaluated the *in vitro* antibacterial activity of similar sealers and found no significant antibacterial activity one day after setting. ^34^ According to Baer *et al.*, sealers without amoxicillin did not inhibit the growth of *E. faecalis.* Additionally, they found no statistical difference between fresh mixed samples and those that had been set (p > 0.05). ^35^ This is in agreement with the present study. Pizzo *et al.* in 2006 evaluated *in vitro* antimicrobial action of root canal sealers and found ZOE sealer to be equally effective in inhibiting bacterial growth until 24 hours after mixing. ^36^ It is likely that the set materials released eugenol that contributed to this. ^37^

The SA group (sealer with antibiotics at 24 hours) combination showed a marked positive inhibitory effect on *E. faecalis.* ( Figure 1 – [c] Baseline, [d] after 24 h) A possible hypothesis is the alkaline pH of the combination enhancing the antimicrobial activity against *E. faecalis* and its susceptibility towards amoxicillin. The results are in agreement with Baer *et al.* who evaluated *in vitro*, the antibacterial effect of amoxicillin when added to different sealers and found that sealers mixed with amoxicillin were significantly more effective than without. ^35^ According to Binoy *et al.*, the reported, pH of the combination was 8.55, this high alkalinity might have deleterious effects on microorganisms in obturated canals. ^37^ Vidulasri *et al.* stated that the anti-microbial activity against *E. faecalis* was improved when antibiotics like amoxicillin and clindamycin were added to zinc oxide eugenol sealer. Amoxicillin is beta-lactam bactericidal broad-spectrum antibiotic that acts by inhibiting bacterial cell wall synthesis. ^29^ According to a previous study, *E. faecalis* is more sensitive to the antibiotics amoxicillin, amoxicillin-clavulanic acid, benzyl penicillin, vancomycin, and doxycycline while being less sensitive to the antibiotics erythromycin and azithromycin. ^38^ An antibiotic-enhanced sealer that alters the environment of the microorganism and retains bactericidal qualities after setting time may be crucial for the success of initial endodontic therapy and for avoiding re-infection. ^39^^,^^40^

The S7 group (sealer without antibiotics after seven days) showed significant improvement in antimicrobial effect compared to S group with a reversal in the green to red ratio. [ Figure 2 – [a] Baseline, [b] after seven days] (p < 0.01). A possible hypothesis is a bactericidal effect of eugenol due to its sustained release on microbes causing protein denaturation. The results are in agreement with Pizzo G *et al*., who found that after seven days from mixing, ZOE containing sealer, still exerted antibacterial activity. ^36^ Heling demonstrated the antibacterial property of Pulp Canal Sealer (EWT) at seven days owing to the bactericidal effect of eugenol as a part of the liquid. ^27^ Hasheminia *et al.* stated that eugenol is a potent antibacterial agent, acting on microbes by protein denaturation. ^41^ The findings of the present study demonstrating antibacterial activity of EWT after seven days are justified by the above mentioned studies. The results are contradicted by Smadi *et al.* who tested nine sealers against *E. faecalis* and found that most sealers had antibacterial properties immediately after mixing, but these properties deteriorated over time. ^42^ Zhang *et al.* also did not see any antibacterial effect of eugenol based sealer beginning on the third day. ^34^ This could be because of the use of different formulations of sealers or use of different microbiological techniques of assessment. In our study results were contrary to these findings probably because of the use of confocal laser scanning microscope for analysis.

The SA showed good antibacterial efficacy in 24 hours which could be because of the presence of antibiotics and maintained its efficacy even after seven days in the SA7 group (sealer with antibiotic after seven days) ( Figure 2 – [c] Baseline, [d] after seven days) with no significant statistical difference with the S7 and SA. The results are in agreement with Baer *et al*. ^35^ who concluded that the antimicrobial properties and inhibition of the *E. faecalis* growth even after seven days can be demonstrated in sealers combined with amoxicillin. However, a slight increase in the viable cell count is seen when compared with the 24 hour group, which could probably be because of multiplication of cell adjacent to the zone of killing or slow release of amoxicillin from a viscous mix. ^43^ Our results are in agreement with Binoy *et al.* who reported the combination to have greater viscosity when compared with other antibiotic combinations. This finding further explains the reason for no enhancement in the antimicrobial property of 10% amoxicillin sealer combination after seven days when compared with 24 hours. ^37^ However, more longitudinal clinical studies are needed to confirm the outcome of addition of different antibiotics to various sealers.

## Conclusions

Within the limitations, the outcome of the current study concluded that adding antibiotics 10% amoxicillin to zinc oxide eugenol sealer increases its antibacterial effect against *E. faecalis* within 24 hours, while plain ZOE sealer start demonstrating good antibacterial property against *E. faecalis* only after seven days.

### Key points

• Adding antibiotics to sealer may help in preventing post obturation infection of the periapical region.
• Since amoxicillin is reported as the drug of choice for endodontic infections in most countries, 10% amoxicillin is added to sealer in the present research, to prevent reinfection. Clindamycin and erythromycin can be alternative drugs for patients allergic to penicillin.
• Adding amoxicillin to ZOE sealer, the antibacterial property of sealer will be improved within 24 h.

## Data Availability

Figshare: master chart thesis.xlsx, https://doi.org/10.6084/m9.figshare.19180589.v1. Data are available under the terms of the Creative Commons Attribution 4.0 International license (CC-BY 4.0).
