# Supplementary material for: Antimicrobial efficacy of Kerr pulp canal sealer (EWT) in combination with 10% amoxicillin on Enterococcus faecalis: A confocal laser scanning microscopic study
Source: F1000Res. 2023 Aug 14;12:725. Originally published 2023 Jun 21. [Version 2] doi: 10.12688/f1000research.132047.2 (PMC10382784; doi:10.12688/f1000research.132047.2)
Supplement: Peer review history

# Peer review: Reviewer response for version 2

**DOI:** 10.5256/f1000research.153815.r196511

**Published:** 2023-08-30

**Authors:** Saravana Karthikeyan Balasubramanian

**Affiliations:** 1 Department of Conservative Dentistry and Endodontics, SRM Dental College, Ramapuram, SRM Institute of Science and Technology, Chennai, Tamil Nadu, India

I have gone through the comments addressed by the authors. The revisions seem to be appropriate and hence, the article can be accepted for indexing in this revised form.

Is the work clearly and accurately presented and does it cite the current literature?

Yes

If applicable, is the statistical analysis and its interpretation appropriate?

Partly

Are all the source data underlying the results available to ensure full reproducibility?

Partly

Is the study design appropriate and is the work technically sound?

Yes

Are the conclusions drawn adequately supported by the results?

Yes

Are sufficient details of methods and analysis provided to allow replication by others?

Partly

Reviewer Expertise:

NA

I confirm that I have read this submission and believe that I have an appropriate level of expertise to confirm that it is of an acceptable scientific standard.

# Peer review: Reviewer response for version 2

**DOI:** 10.5256/f1000research.153815.r196510

**Published:** 2023-08-21

**Authors:** Arindam Dutta

**Affiliations:** 1 School of Dentistry, College of Biomedical and Lifesciences, Cardiff University, Cardiff, UK

No further comments.

Is the work clearly and accurately presented and does it cite the current literature?

Yes

If applicable, is the statistical analysis and its interpretation appropriate?

Yes

Are all the source data underlying the results available to ensure full reproducibility?

Partly

Is the study design appropriate and is the work technically sound?

Partly

Are the conclusions drawn adequately supported by the results?

Yes

Are sufficient details of methods and analysis provided to allow replication by others?

Yes

Reviewer Expertise:

Endodontics, Restorative Dentistry

I confirm that I have read this submission and believe that I have an appropriate level of expertise to confirm that it is of an acceptable scientific standard.

# Peer review: Reviewer response for version 1

**DOI:** 10.5256/f1000research.144944.r181658

**Published:** 2023-07-28

**Authors:** Arindam Dutta

**Affiliations:** 1 School of Dentistry, College of Biomedical and Lifesciences, Cardiff University, Cardiff, UK

Many thanks to the authors for submitting this paper to the journal. The paper has used some sophisticated techniques to assess dead/live bacteria in response to root canal treatment procedures. However, the premise for the paper: using an antibiotic in a sealer does not represent a contemporary approach. Root canal infections have multiple micro-organisms and an antibiotic like amoxicillin alone will not suffice. Secondly, the experimental model used dentine which had a mono-infection of *E. faecalis*, which also does not represent the clinical scenario - a multispecies biofilm has been proposed for such *in vitro* studies. Nevertheless, as an initial step, in trying to understand whether an antibiotic may have some influence on *E.faecalis* mono-infection, this is an acceptable investigation which has shown the lack of benefit. This helps establish that further studies in this direction are not warranted.

Is the work clearly and accurately presented and does it cite the current literature?

Yes

If applicable, is the statistical analysis and its interpretation appropriate?

Yes

Are all the source data underlying the results available to ensure full reproducibility?

Partly

Is the study design appropriate and is the work technically sound?

Partly

Are the conclusions drawn adequately supported by the results?

Yes

Are sufficient details of methods and analysis provided to allow replication by others?

Yes

Reviewer Expertise:

Endodontics, Restorative Dentistry

I confirm that I have read this submission and believe that I have an appropriate level of expertise to confirm that it is of an acceptable scientific standard, however I have significant reservations, as outlined above.

# Response

**Published:** 2023-07-29

**Authors:** Kundabala Mala

**Affiliations:** Conservative Dentistry, Manipal Academy of Higher Education, Manipal, Karnataka, India

Thanks a lot for the respected reviewer for the pragmatic inputs.  The premise for the paper: using an antibiotic in a sealer does not represent a contemporary approach.

Answer: A series of studies are following. Invitro multiple microbic study will be slightly difficult at this moment since invitro conducive growth environment have to be created for each organism.

 Query: Root canal infections have multiple micro-organisms and an antibiotic like amoxicillin alone will not suffice.

Answer: Penicillin VK and amoxicillin, both beta-lactam antibiotics, are the first line of antibiotics chosen as adjunct therapeutic agents in endodontics, according to AAE Guidance on the Use of Systemic Antibiotics in Endodontics. Hence we used in our study.

 Secondly, the experimental model used dentine which had a mono-infection of *E. faecalis*, which also does not represent the clinical scenario - a multispecies biofilm has been proposed for such *in vitro* studies.

 Amoxicillin demonstrates greater efficacy and therapeutic value even in multispecies endodontic infections. This drug acts by binding and inhibiting the activity of several bacterial proteins nvolved in the synthesis of the peptidoglycan cell wall in susceptible both gram-positive and gram-negative bacteria (23). This drug has been found to be highly effective against isolates from infected root canal systems that are composed primarily of facultative and obligate anaerobes. It is representation for multispecies since it is one of the most drug resistant organism for antibacterial agents used in endodontics. Hence for convenience we used single organism in our study.  

 Thanks again for these good suggestions.

# Peer review: Reviewer response for version 1

**DOI:** 10.5256/f1000research.144944.r181661

**Published:** 2023-07-27

**Authors:** Moksha Nayak

**Affiliations:** 1 Department of Conservative Dentistry & Endodontics, KVG Dental College & Hospital, Sullia, Karnataka, India

Disinfection of root canal system is a most important step for the success of endodontictherapy. During instrumentation irrigants and intracanal medicaments are used. Adding antibiotics to sealers is beneficial to prevent re-infection and impart antimicrobial property for an extended period of time at the post-endodontic period.

 The work done by the authors is contemporary and need of the hour. The manuscript shows the study will be clearly understood by the readers and accurately presented. Regarding the citation of the current literature, I appreciate this manuscript is focused on synergizing the effect of these sealers by adding antibiotics to the sealer and its effect by studying their antimicrobial efficacy on microbes by one of the latest technologies-Confocal Laser Microscopy and most of the previous studies are done using culture and other older techniques. Moreover, live and dead bacterial status also has been studied. There are no sealer-antibiotics studies recently done using CLSM or any other recent technology trying to explore actual bacterial status of this combination.

 Proper background for the study is established. Adequate justification is given for selecting this research question in the introduction. In the abstract section some corrections can be done regarding grammatical errors.

 Abstract:

 Conclusion: “Antibiotics added to the sealer effectively eradicated of E. faecalis 24 hours post-obturation.”.

 In the Materials and method section sample size is adequate and methodology is properly formulated and it ensures full reproducibility. Results are well documented and discussed well. All the sources of data underlying the results are available .

 Conclusions drawn are adequately supported by the results.

 The following are my suggestions after going through the article:

- There are a few more papers published in the literature comparing various antibiotic-sealer combinations on endodontic microbes. Recent ones have studied the antibiotic combinations such as Triple (TAP) or Double antibiotic paste (DAP).
- The authors could have included TAP or DAP combination with epoxy resin-based sealers or bioceramic sealers. Could the authors justify why they have not considered these options?
   
  - Kangarlou *et al.* (2016 ^1^).
  - Brezhnev *et al. *(2019 ^2^).
- In key points 2, Clindamycin and erythromycin can be alternative drugs for patients allergic to penicillin. The study has not included these antibiotics. Hence the authors need not mention about these.
- A focused review on antibacterial additives in epoxy resin-based root canal sealers by Brezhnev *et al.* (2019 ^2^) could have been added in the discussion to justify the use of CLSM in the study.

Is the work clearly and accurately presented and does it cite the current literature?

Yes

If applicable, is the statistical analysis and its interpretation appropriate?

Yes

Are all the source data underlying the results available to ensure full reproducibility?

Yes

Is the study design appropriate and is the work technically sound?

Yes

Are the conclusions drawn adequately supported by the results?

Yes

Are sufficient details of methods and analysis provided to allow replication by others?

Yes

Reviewer Expertise:

NA

I confirm that I have read this submission and believe that I have an appropriate level of expertise to confirm that it is of an acceptable scientific standard.

## References

[1] : Antibacterial efficacy of AH Plus and AH26 sealers mixed with amoxicillin, triple antibiotic paste and nanosilver. *J Dent Res Dent Clin Dent Prospects* .2016;10(4) : 10.15171/joddd.2016.035 220-225 10.15171/joddd.2016.035 28096947PMC5237668

[2] : Antibacterial Additives in Epoxy Resin-Based Root Canal Sealers: A Focused Review. *Dent J (Basel)* .2019;7(3) : 10.3390/dj7030072 10.3390/dj7030072 31266165PMC6784478

# Response

**Published:** 2023-08-12

**Authors:** Kundabala Mala

**Affiliations:** Conservative Dentistry, Manipal Academy of Higher Education, Manipal, Karnataka, India

- The authors could have included TAP or DAP combination with epoxy resin-based sealers or bioceramic sealers. Could the authors justify why they have not considered these options?
- Authors' reply: Amoxicillin has been proved already to be one of the best antibiotagainst endodontic microbes. Hence we tried as additives to the sealer. However, we plan to conduct our future studies with TAP or DAP
J. J. Segura-Egea, K. Gould, B. Hakan Şen, P. Jonasson, E. Cotti, A. Mazzoni, H. Sunay, L. Tjäderhane, P. M. H. Dummer.Antibiotics in Endodontics: a review.International Endodontic Journal . Volume 50, Issue12 December 2017 Pages 1169-1184
- In key points 2, Clindamycin and erythromycin can be alternative drugs for patients allergic to penicillin. The study has not included these antibiotics. Hence the authors need not mention about these.
- Authors' reply: We have put it as recommendation and suggestions to the readers if the patient is allergic to amoxicillin; not the conclusion of our study.
- A focused review on antibacterial additives in epoxy resin-based root canal sealers by Brezhnev *et al.* (2019 ^2^ ) could have been added in the discussion to justify the use of CLSM in the study.
- Authors' reply: It is future scope of our study.

# Peer review: Reviewer response for version 1

**DOI:** 10.5256/f1000research.144944.r181659

**Published:** 2023-07-18

**Authors:** Saravana Karthikeyan Balasubramanian

**Affiliations:** 1 Department of Conservative Dentistry and Endodontics, SRM Dental College, Ramapuram, SRM Institute of Science and Technology, Chennai, Tamil Nadu, India

This interesting clinically significant study compared the antibacterial efficacy of a zinc oxide eugenol based sealer with and without 10% amoxicillin combination against *E.faecalis*. However, this manuscript requires minor modifications before acceptance for indexing.

 Please find my comments attached herewith:

**Comments:**

**Page 1, Abstract:** 

 Background, Line 2: " *Enterococcus faecalis*" and its abbreviation " *E.faecalis*" are used alternatively. It is recommended that the full form of the bacteria can be mentioned initially, followed by subsequent mention of its abbreviation.

 Methods, Line 4: Please mention the use of 17% EDTA during canal instrumentation.

 Results, Line 4: It is highly appreciable to mention the mean and SD values as well along with the statistical results.

 Conclusions, Line 1: The conclusions can be very specific, addressing the effect of 10% amoxycillin rather than the mention of “Antibiotics” against *E.faecalis*.

 Based on the derived conclusions, the authors can make recommendations for inclusion of antibiotics to root canal sealers for potential eradication of the bacteria.

**Page 2, Introduction:** 

 As the effect of the combination of 10% amoxycillin and Kerr Pulp Canal Sealer (EWT) against *E.faecalis*, has been previously studied by Hoelscher *et al.*, (2006[ref-1), I would recommend the authors to highlight the strong rationale of the current investigation before addressing the aim.

 (Please refer to: Hoelscher *et al.*, 2006 ^1^).

 Null/alternative hypothesis also must be mentioned.

**Page 2, Methods:** 

 How was the sample size of 60 arrived to? Details mentioning the pilot study or power calculation based on previous literature studies can be highlighted.

 Inclusion and exclusion criteria of the extracted teeth including, the reasons for extraction, patient age group, canal curvature etc., can be mentioned.

**Page 2, Methods:** 

 Infection of dentin blocks: In the present study, the dentinal blocks were infected with *E.faecalis* for 4 days. However, literature evidence supports 4-weeks infection time period to ensure adequate penetration of *E.faecalis* into dentinal tubules.

 Please refer to:

- Haapsalo and Ørstavik (1987 ^2^).
- Shabahang and Torabinejad (2003 ^3^).

**Page 8 & 9, Discussion:** 

 The authors can highlight the virulence factors of *E.faecalis* substantiating the reason why *E,.faecalis* and the particular strain was studied?

 Role of NaOCl in inhibiting *E.faecalis* should also be discussed by the authors, which is clinically relevant. In the current study, 5.25% NaOCl was employed. There are contraindicatory findings reported in literature when different concentrations of NaOCl were tested against *E,faecalis*. The authors are encouraged to shed light on these observations as well. (Please refer to: Shabahang and Torabinejad (2003 ^3^)).

 Advantages of CLSM method can be highlighted. The authors can discuss in view of the methodology employed by Hoelscher *et al.,* (2006[ref-1) who earlier tested the efficacy of 10% amoxicillin-Kerr Pulp canal Sealer EWT combination using agar diffusion method.

 Based on the results obtained, the authors can either accept or reject the proposed null hypothesis.

 Further, the limitations and scope of the study should be highlighted. Limitations of the study may include sample size and infection time period. Future research investigations pertaining to the evaluation of the sealing properties such as shrinkage, setting time, diffusability and substantivity of the sealer-antibiotic combination needs detailed analysis. In addition, the antibiotic resistance and allergic host response to the antibiotic formulation also needs careful exploration.

**General Comments:**

 The authors are requested to check the language throughout the length of the manuscript. For instance: Page 1, Abstract: Methods section - “Root canal standardized to 12 mm”; Page 2, Keywords: “Obturaion”; Page 3, Introduction, Line 3: “This can be achieved through…. Chemical preparation mechanical preparation…….”- Can be mentioned as “chemo-mechanical preparation”.

 Please mention the full form of technical terms, when they are cited in text for the first time, subsequently followed by the mention of their abbreviations. (e.g., *E.faecalis*, CLSM, NaOCl, EDTA etc.,).

Is the work clearly and accurately presented and does it cite the current literature?

Yes

If applicable, is the statistical analysis and its interpretation appropriate?

Partly

Are all the source data underlying the results available to ensure full reproducibility?

Partly

Is the study design appropriate and is the work technically sound?

Yes

Are the conclusions drawn adequately supported by the results?

Yes

Are sufficient details of methods and analysis provided to allow replication by others?

Partly

Reviewer Expertise:

Endodontics, Irrigants, Disinfection, Dentin Tissue Engineering, Biomaterials

I confirm that I have read this submission and believe that I have an appropriate level of expertise to confirm that it is of an acceptable scientific standard, however I have significant reservations, as outlined above.

## References

[1] : In vitro evaluation of the antimicrobial effects of a root canal sealer-antibiotic combination against Enterococcus faecalis. *J Endod* .2006;32(2) : 10.1016/j.joen.2005.10.031 145-7 10.1016/j.joen.2005.10.031 16427465

[2] : In vitro infection and disinfection of dentinal tubules. *J Dent Res* .1987;66(8) : 10.1177/00220345870660081801 1375-9 10.1177/00220345870660081801 3114347

[3] : Effect of MTAD on Enterococcus faecalis-contaminated root canals of extracted human teeth. *J Endod* .2003;29(9) : 10.1097/00004770-200309000-00008 576-9 10.1097/00004770-200309000-00008 14503830

# Response

**Published:** 2023-07-28

**Authors:** Kundabala Mala

**Affiliations:** Conservative Dentistry, Manipal Academy of Higher Education, Manipal, Karnataka, India

Please find my comments attached herewith:

Authors: Thanks a lot respected reviewer for going through the article in detail and giving scientific and very encouraging comments.

**Comments:**

**Page 1, Abstract:** 

 Query: Background, Line 2: " *Enterococcus faecalis*" and its abbreviation " *E.faecalis*" are used alternatively. It is recommended that the full form of the bacteria can be mentioned initially, followed by subsequent mention of its abbreviation.

Answer: It is done in resubmitted manuscript.

 Query: Methods, Line 4: Please mention the use of 17% EDTA during canal instrumentation.

Answer: 17% EDTA is used for removing smear layer generated during instrumentation.

 Results, Line 4: It is highly appreciable to mention the mean and SD values as well along with the statistical results.

Answer: The mean and standard deviations between green ratio, red ratio, green to red ratio and p values for  all the four groups are mentioned in Table 1.

 Query: Conclusions, Line 1: The conclusions can be very specific, addressing the effect of 10% amoxycillin rather than the mention of “Antibiotics” against *E.faecalis*.

Answer: Adding 10% amoxicillin to Kerr pulp canal sealer (EWT) against E. Faecalis may help in preventing post obturation infection of the periapical region.

 Query: Based on the derived conclusions, the authors can make recommendations for inclusion of antibiotics to root canal sealers for potential eradication of the bacteria.

Answer: Conclusion is modified to 10% amoxicillin added to the Kerr pulp canal sealer (EWT) effectively eradicated of E. faecalis at 24 hours post-obturation. However, after seven days, plain sealer was as effective as sealer-antibiotic combination. Based on the derived conclusions, it can be recommended that inclusion of antibiotics to root canal sealers help in  eradication of the bacteria.

**Page 2, Introduction:** 

 As the effect of the combination of 10% amoxycillin and Kerr Pulp Canal Sealer (EWT) against *E.faecalis*, has been previously studied by Hoelscher *et al.*, (2006[ref-1), I would recommend the authors to highlight the strong rationale of the current investigation before addressing the aim.

 (Please refer to: Hoelscher *et al.*, 2006 ^1^).

It is done in the resubmitted manuscript.

 Query: Null/alternative hypothesis also must be mentioned.

Answer: The null hypothesis of the study is there is no difference in the antimicrobial efficacy of the Kerr pulp canal sealer (EWT) with or without addition of 10% amoxicillin against *E. faecalis,*

**Page 2, Methods:** 

 Query: How was the sample size of 60 arrived to? Details mentioning the pilot study or power calculation based on previous literature studies can be highlighted.

Answer: Using G*Power 3.1.9.4 software, at 95% confidence level, 90% power, 0.5 effect size, and assuming 20% loss of sample during the process, the sample size for the present study was calculated to be 60 in each group.

 Query: Inclusion and exclusion criteria of the extracted teeth including, the reasons for extraction, patient age group, canal curvature etc., can be mentioned.

 Answer:

Inclusion criteria:

freshly extracted single rooted human premolars with Type I canal anatomy and mature root apex extracted for orthodontic purpose from patients of age group 18-25  selected

Exclusion criteria:

Fractured teeth, immature open apex, curved roots, root canals other than Type-I canal anatomy

**Query: Page 2, Methods:** 

 Infection of dentin blocks: In the present study, the dentinal blocks were infected with *E.faecalis* for 4 days. However, literature evidence supports 4-weeks infection time period to ensure adequate penetration of *E.faecalis* into dentinal tubules.

 Please refer to:

- Haapsalo and Ørstavik (1987 ^2^).
- Shabahang and Torabinejad (2003 ^3^ ).

Answer: The present study followed the protocol suggested by  Flaviana Bombarda de ANDRADE et. al. (REF.28) for in vitro intratubular dentinal bacterial contamination for antimicrobial endodontic tests for confocal laser scanning microscopy.

**Page 8 & 9, Discussion:** 

**Query: The authors can highlight the virulence factors of E.faecalis substantiating the reason why E,.faecalis and the particular strain was studied?**

**Answer: E. faecalis was chosen as study microbe since it is found especially in failed to endodontic infection s. It is because of its virulence factors such as aggregation substance, surface adhesins, sex pheromones, lipoteichoic acid, extracellular superoxide production, the lytic enzymes gelatinase and hyaluronidase, and the toxin cytolysin .REF: 32. The present study used CLSM for evaluating antimicrobial efficacy od sealer since it is one of the most commonly used fluorescence microscopic techniques recently in endodontics, particularly in three-dimensional (3D) studies involving biological cells and tissues. It is a flexibil e approach , which makes it suitable for use in fast imaging of dynamic processes in living cells, sensitive morphological analysis of tissues . REF: 3 3**

**The Reasons or choosing the  E. Faecalis  as study microbe s already mentioned in the introduction, all the incorporated  in the resubmitted manuscript**

 Query: Role of NaOCl in inhibiting *E.faecalis* should also be discussed by the authors, which is clinically relevant. In the current study, 5.25% NaOCl was employed. There are contraindicatory findings reported in literature when different concentrations of NaOCl were tested against *E,faecalis*. The authors are encouraged to shed light on these observations as well. (Please refer to: Shabahang and Torabinejad (2003 ^3^)).

 Answer:

 Sodium hypochlorite possesses an effective antibacterial action against *E. faecalis* its level of effectiveness depends mainly upon a)its concentration and form, b) different models of the microorganism and c) experimental procedures including manipulation and duration of contact with the microorganism

 Ref: Luddin N, Ahmed HM. The antibacterial activity of sodium hypochlorite and chlorhexidine against Enterococcus faecalis: A review on agar diffusion and direct contact methods. J Conserv Dent. 2013 Jan;16(1):9-16.. 

 Advantages of CLSM method can be highlighted. The authors can discuss in view of the methodology employed by Hoelscher *et al.,* (2006[ref-1) who earlier tested the efficacy of 10% amoxicillin-Kerr Pulp canal Sealer EWT combination using agar diffusion method.

Advantages of CLSM are  included in the discussion. Corrections are incorporated  in the resubmitted manuscript.

 Query: Based on the results obtained, the authors can either accept or reject the proposed null hypothesis.

Answer:Based on the results of the present study,authors reject the null hypothesis  and conclude that addition of amoxicillin to sealersimproves the antibacterial efficacy  of the Zinc oxide sealer. The corrections are incorporated  in the resubmitted manuscript.

 Query:Further, the limitations and scope of the study should be highlighted. Limitations of the study may include sample size and infection time period. Future research investigations pertaining to the evaluation of the sealing properties such as shrinkage, setting time, diffusability and substantivity of the sealer-antibiotic combination needs detailed analysis. In addition, the antibiotic resistance and allergic host response to the antibiotic formulation also needs careful exploration.

Answer: Corrections are incorporated in the resubmitted manuscript.

**General Comments:**

 The authors are requested to check the language throughout the length of the manuscript. For instance: Page 1, Abstract: Methods section - “Root canal standardized to 12 mm”; Page 2, Keywords: “Obturaion”; Page 3, Introduction, Line 3: “This can be achieved through…. Chemical preparation mechanical preparation…….”- Can be mentioned as “chemo-mechanical preparation”.

 Please mention the full form of technical terms, when they are cited in text for the first time, subsequently followed by the mention of their abbreviations. (e.g., *E.faecalis*, CLSM, NaOCl, EDTA etc.,).

Corrections are incorporated  in the resubmitted manuscript.

- **Is the work clearly and accurately presented and does it cite the current literature?**

Yes

- **Is the study design appropriate and is the work technically sound?**

Yes

- **Are sufficient details of methods and analysis provided to allow replication by others?**

Partly

- **If applicable, is the statistical analysis and its interpretation appropriate?**

Partly

- **Are all the source data underlying the results available to ensure full reproducibility?**

Partly

- **Are the conclusions drawn adequately supported by the results?**

Yes

**References**

 1. Hoelscher AA, Bahcall JK, Maki JS: In vitro evaluation of the antimicrobial effects of a root canal sealer-antibiotic combination against Enterococcus faecalis. *J Endod*. 2006; **32** (2): 145-7 PubMed Abstract | Publisher Full Text

 2. Haapasalo M, Orstavik D: In vitro infection and disinfection of dentinal tubules. *J Dent Res*. 1987; **66** (8): 1375-9 PubMed Abstract | Publisher Full Text

 3. Shabahang S, Torabinejad M: Effect of MTAD on Enterococcus faecalis-contaminated root canals of extracted human teeth. *J Endod*. 2003; **29** (9): 576-9 PubMed Abstract | Publisher Full Text
